# Outdoor recreation, tick borne encephalitis incidence and seasonality in Finland, Norway and Sweden during the COVID-19 pandemic (2020/2021)

**DOI:** 10.1080/20008686.2023.2281055

**Published:** 2023-11-18

**Authors:** Solveig Jore, Hildegunn Viljugrein, Marika Hjertqvist, Timothée Dub, Henna Mäkelä

**Affiliations:** a Zoonotic, Food & Waterborne Infections, Norwegian Institute of Public Health (NIPH), Oslo, Norway; b Norwegian Veterinary Institute, Norway; c Centre for Ecological and Evolutionary Synthesis (CEES), Department of Biosciences, University of Oslo, Blindern, Norway; d Department of Communicable Disease Control and Health Protection, Public Health Agency of Sweden, Solna, Sweden; e Infectious Disease Control and Vaccinations Unit, Department of Health Security, Finnish Institute for Health and Welfare (THL), Helsinki, Finland

**Keywords:** TBE, tick-borne encephalitis, seasonality, public health interventions, COVID-19, COVID-19 restrictions

## Abstract

During the pandemic outdoor activities were encouraged to mitigate transmission risk while providing safe spaces for social interactions. Human behaviour, which may favour or disfavour, contact rates between questing ticks and humans, is a key factor impacting tick-borne encephalitis (TBE) incidence.

We analyzed annual and weekly TBE cases in Finland, Norway and Sweden from 2010 to 2021 to assess trend, seasonality, and discuss changes in human tick exposure imposed by COVID-19. We compared the pre-pandemic incidence (2010–2019) with the pandemic incidence (2020–2021) by fitting a generalized linear model (GLM) to incidence data.

Pre-pandemic incidence was 1.0, 0.29 and 2.8 for Finland, Norway and Sweden, respectively, compared to incidence of 2.2, 1.0 and 3.9 during the pandemic years. However, there was an increasing trend for all countries across the whole study period. Therefore, we predicted the number of cases in 2020/2021 based on a model fitted to the annual cases in 2010–2019. The incidences during the pandemic were 1.3 times higher for Finland, 1.7 times higher for Norway and no difference for Sweden. When social restrictions were enforced to curb the spread of SARS-CoV-2 there were profound changes in outdoor recreational behavior. Future consideration of public health interventions that promote outdoor activities may increase exposure to vector-borne diseases.

## Introduction

Tick-borne encephalitis (TBE) virus is a member of the flavivirus-family that can cause meningitis, encephalitis and/or radiculitis. The first description of a tick-borne encephalitis-like disease in the central part of western Europe is still debated and uncertain. In the Nordic countries, the seemingly first official reports of TBE from Sweden, Finland, Denmark and Norway were in 1925, 1956, 1963 and 1997, respectively [1–3]. Ticks act as both a vector and reservoir of the TBE-virus (TBEV). However, collection and screening of ticks by real-time RT-PCR is not a sensitive indicator for risk assessment of tick-borne encephalitis in humans [4]. The main vector for the European subtype of the virus (TBEV-Eu) in Central, Eastern and Western Europe is *Ixodes ricinus*. Humans are only accidental viral hosts, with small-to-medium sized mammals functioning as reservoir hosts. Little is currently known about tick-borne virus persistence in wild vertebrates in natural populations [5,6]. Small mammalian animals are often heavily infested with juvenile ticks and are presumed to be competent virus reservoir hosts [6,7]. There is however a lack of studies of small mammals as classic virus reservoirs for TBEV [8–10]. A striking feature is the prominent focal distribution of TBEV and consequently the risk of infection restricted to small foci [11]. TBE incidence has increased in Europe over the past decade [12], although a few countries have experienced a decrease during the same period. A newly published paper show that there has been a statistically significant increase in TBE cases during 2012–2020 in EU/EEA countries with an average of 0.053 additional TBE cases per week [12]. The drivers and mechanisms are not understood except for a chain of biotic, abiotic and human-induced impacts on all components of the disease system (pathogen, vector, vertebrate wildlife, and humans) [13].

Known factors that impact TBE incidence can be grouped into different categories: tick abundance, host population dynamics and population at risk [14–18]. Tick abundance and TBE prevalence in ticks are linked to numerous ecological and environmental conditions, like land use, amount and type of forest, climate, and host reservoir species and availability [19–21]. The TBE incidence is associated with several climatic factors [22–25], forested habitats [25–27], climate change [28], host population dynamics [18,23,29,30] and different behavioural and sociological factors [31–34]. The amount of forest or forest type might indirectly also reflect human habits (mushrooms and berries picking) and not only host community composition/size and exposure to ticks in the understory. Notably, the scientific reasoning behind forecast of TBE incidence made for central European countries [35–37] cannot be extrapolated to Scandinavian countries since beech forests are not commonly found in Finland, Norway and Sweden. However, human behaviour, which may favour or disfavour contact rates between questing ticks and humans is regarded as a key factor influencing TBEincidence [34] and was ranked highest in the first expert elicitation of possible drivers [13]. This adds complexity and instability to the spatio-temporal dynamics of these disease systems. Here, climate or weather also play an indirect role, as favourable weather conditions promote outdoor activities [38]. An example is the unusually high TBE numbers seen in some European countries in 2006 that were explained by changes in recreational behaviour of humans; people spending more time outdoors during this extremely warm year [39]. Outdoor activities also depend on spatial factors such as forest cover and tourism development. Additionally, socio-economic factors might increase uptake of certain outdoor risk activities [38,40], leading to further exposure. The type of eco-social setting (natural areas, urban green spaces or peridomestic environments) can potentially also interact and impact the disease risk [41–43]. The vaccination uptake, completion and compliance in endemic areas has a decisive effect on TBE incidence. TBE vaccination data is not available for all three countries; so, we do not know who has been vaccinated and where.

TBE is a notifiable disease in Finland, Norway and Sweden. Annually, 60–80 cases are usually reported to the National Infectious Disease Register [44] in Finland, where laboratory confirmed cases have been notifiable since 1995. Over the past years, changes in the spatial distribution of the TBE virus in Finland have been observed [45–47], as the TBE risk-areas have expanded from the south-west coast and archipelago of Åland to the west coast towards Helsinki capital-areas and North as high as the municipality of Kemi. Also, since 2006, when Åland was added to the National Immunization program, the risk-areas have expanded to cover some of the lake-areas in continental Finland in addition to coastal areas. The risk areas for TBE are, with some exceptions, relatively limited and located mostly to the southwestern part of the country [48]. In Norway, human cases are reported by physicians and laboratories to the Norwegian Surveillance System for Communicable Diseases (MSIS) [49] and TBE has been notifiable since 1975. The annual number of reported cases of TBE in Norway usually ranged between 5 and 16 cases, but from 2018 there has been an increasing trend [49] and lately approaching 70–80 cases. All the human cases, and thus the main risk area, are localized to a few counties along the coast of Southern Norway [50]. In Sweden, human TBE cases are reported by physicians and laboratories to the Public Health Agency. TBE has been a notifiable disease since 1969 and at that time about 10–40 cases were reported each year [51]. Since the 1980s the annual incidence of human TBE has increased almost continuously and during the last decade about 200 to 500 cases have been reported annually. Most people have historically been infected on the east coast of Sweden and in the Stockholm archipelago but in recent decades the disease has spread westwards and nowadays it is regularly observed on the west coast of the country as well. The infection at present occurs from the region of Skåne in the south to the regions of Gävleborg and Dalarna in the north of Sweden [52].

COVID-19 restrictions were implemented in all three countries throughout 2020. The Finnish government declared a state of emergency due to the emergence of SARS-CoV-2 throughout the whole country on 16th of March 2020, after which restrictions were implemented in a stepwise fashion and applied to a high degree during the year. Schools were closed, gatherings restricted, public spaces and sports venues were closed, and people were instead encouraged to spend more time outdoors. In Norway on 12 March 2020, the government announced a series of restrictive measures prohibiting mass gatherings, closure of schools, universities, and businesses. However, a strict stay-at-home order was never imposed in either Finland or Norway. Sweden’s response to the pandemic was less restrictive compared to the neighbouring countries and was largely based on voluntary action, urging personal responsibility instead of implementing strict closures. The authorities gave recommendations to stay home if having symptoms that could be due to COVID-19, keep distance from other people, avoid traveling by public transport, shopping, and visiting other crowded places. Unlike in many other countries, schools and day care centres were kept open throughout the pandemic in Sweden.

The pandemic mitigation measures varied across the globe and in countries which enforced stay-at-home orders, where people were obliged to stay indoors (as in Taiwan and China), they saw a general decrease in all faecal-oral, vector-borne and direct-contact transmitted diseases. Especially, infectious diseases caused by air-borne pathogens decreased widely during the period where strict social measures such as mask and stay-at-home orders mandates were in place. In China, the average yearly incidence and mortality rates of vector-borne diseases declined with 72.95% and 77.60%, respectively, in 2019/2020 [53] and a similar decrease was seen in Taiwan [54]. Poland and Estonia registered a significant decrease in TBE incidence (−42,3% and −17,4%) in 2020 [55,56]. Poland imposed a hard stay-at-home order from March to April 2020, with for instance a ban on access to forests and parks, which might have reduced the exposure to tick bites and caused significant reduction in TBE incidence [57]. The decreased incidence (or parts of it) might also been influenced by less reporting by public health officials due to demands related to the pandemic, as suggested for Poland [55]. In Finland, Norway and Sweden, while the reported TBE cases increased, a profound reduced incidence of many infectious diseases was seen during the pandemic years [58–60]. Other European countries saw similar changes: Switzerland and Germany reported 2020 as a record high year of TBE while observing a significant decrease in various other infectious diseases [56,61]. The change in the reported number of TBE cases can be hypothesized to be linked to the large-scale behavioral change imposed by the COVID-19 pandemic [62], as also suggested by others [63,64]. What complicates the analyses is that the pre-pandemic years are not comparable to the post-pandemic years since the pandemic has transformed the office forever and continues to reshape work and our outdoor recreation habits [65–68]. Activities for which most workers expect changes are working more from home and spend more time outdoors [67,68].

In this paper, we describe and analyse data from the National Infectious Disease Register (NIDR) in Finland, SmiNet in Sweden, and MSIS in Norway from 2010 to 2022 comparing the pandemic years with previous years, investigating seasonal patterns, and discussing the potential effects from the COVID-19 restrictions on human behaviour, TBE-trend and seasonality.

## Methods

### Dataset

We used case counts of TBE notified to the national registers between 2010 and 2022. In Finland, an acute laboratory-confirmed TBE case is defined as a patient without previous TBEV infection with coherent central nervous system symptomatology and TBEV-specific antibodies detected in either cerebrospinal fluid or serum based on ECDC guidelines. In Norway, TBE is mandatory to report to the Norwegian Surveillance System for Communicable Diseases (MSIS). All cases are laboratory confirmed (TBE-specific IgM and IgG in serum and/or spinal fluid). In Sweden, a national electronic surveillance system (SmiNet) has been in place since 1997 and TBE has been reported there from both clinicians and laboratories since 2004. All cases are laboratory confirmed by either the detection of TBEV-specific IgM antibodies in serum and absence of TBEV-specific IgG antibodies in serum (probable case) or a TBEV-specific antibody response or TBEV viral nucleic acid (confirmed case).

### Data analysis

#### Annual TBE incidence

Annual cases and incidences (per 100 000 persons) were summarized for each country. The risk of TBE infection is linked to specific regions within the three countries, and the spatial distribution of TBE risk has changed over years. The actual population size at risk for TBE infection in the three countries is unknown, for an approximation we used the total annual population size in the respective country to estimate incidence. Annual cases and incidences of pre-pandemic years (2010–2019) was compared with annual cases and incidences during the pandemic years (2020–2021). To test whether the incidence during the pandemic years were significantly different from the incidence in the pre-pandemic years, we fitted a generalized linear model (GLM) with negative binomial distribution to the annual cases (to account for overdispersion). The regression model included a linear trend effect (at log-scale) of year to account for autocorrelation in the time series. The model was utilized to show estimated (2010–2019) and predicted levels (for 2020–2022 and the two-year average of the pandemic years), including 95% confidence intervals, based on the model fitted to the 2010–2019 period. Annual incidences were modelled by including an offset of the annual log population size in the model. If annual reported cases (incidences) from the pandemic years (and 2022) were higher than the predicted 95% confidence intervals, they were considered significantly higher than the pre-COVID-19 years, even when accounting for the long-term increasing trend. P-values were extracted by fitting the regression model to the time period 2010–2021 and in addition to year, including a categorical variable to identify the pandemic years.

#### TBE seasonality

Seasonal patterns were investigated by descriptive summary statistics of weekly reported cases of TBE (2010–2021), emphasising seasonal characteristics of the annual TBE dynamics, such as onset of season and peak weekly cases. We used the first week in a year with at least 2 cases in total reported for the present and previous week to define the onset of the TBE season. For each year, we extracted the peak number of weekly cases and the corresponding week number for the first and last occurrence of the peak (equal, if maximum number of reported cases was reached in only one week of the year). Differences in the descriptive annual summary statistics of the weekly TBE cases between the years 2010–2019 and the COVID-19 years (2020–2021) were tested by using a GLM with quasi-Poisson distribution to account for over (or under) dispersion in the data. The model included a categorical variable to identify the pre-pandemic years from the pandemic years. For extracting the main seasonal pattern of TBE across years, we fitted for each country a seasonal mixed effect GLM with Poisson distribution of weekly reported cases. The model was utilized to separate the seasonal trend from the long-term annual trend in the data. The model was fitted in R-INLA [69], using a cyclic (restricted to start and end at the same level each year) random walk function to fit the seasonal trend (allows for a flexible spline with one knot per week of a year), a first-order random walk function for modelling the long-term trend over years, and modelling overdispersion by using an autoregressive function of order one to account for remaining autocorrelation (for more details, including R-code example and definition of model parameters, see supplementary material to [70]). The full seasonal model is a combination of a long-term annual trend, the seasonal trend (common across all years) and a part accounting for remaining autocorrelation. The seasonal trends were compared between the three countries. All analyses were performed in R version 4.2.1 [71].

## Results

### Annual TBE incidence

During the pandemic years (2020 and 2021) the mean yearly reported cases were 121 (91 and 151) for Finland, 57 (41 and 72) for Norway and 405 (276 and 533) for Sweden compared to the mean yearly reported cases between 2010 and 2019 (Table 1). The mean number of reported TBE cases for 2020–2021 increased by 113% (Finland), 274% (Norway) and 46% (Sweden) Similarly, for the pandemic years, the mean annual incidence per 100 000 inhabitants were 2.2, 1.0 and 3.9, for Finland, Norway and Sweden, respectively, while the mean annual incidence (interquartile range in parenthesis) for 2010–2019 were 1.0 (0.7–1.2), 0.29 (0.19–0.30) and 2.8 (2.2–3.3) for Finland, Norway and Sweden (Table 1). Mean incidence increased by 111% (Finland), 260% (Norway) and 38% (Sweden) during the pandemic years.

**Table 1.** Annual reported TBE (a) cases and (b) incidences from Finland, Norway and Sweden, summarized for pre-pandemic and covid-19 years.

	Pre-pandemic years 2010–2019						COVID-19 years			After COVID
	Mean	Min	25%	50%	75%	Max	Mean	2020	2021	2022
*a) Annual number of cases*										
Finland	56.7	38	40	54	68.8	85	121	91	151	123
Norway	15.1	6	9.5	12.5	15.5	37	56.5	41	72	69
Sweden	277.1	175	216.2	275.5	339.5	391	404.5	276	533	467
*b) Annual incidence*										
Finland	1.0	0.7	0.7	1.0	1.2	1.5	2.2	1.6	2.7	2.2
Norway	0.3	0.1	0.2	0.2	0.3	0.7	1.0	0.8	1.3	1.3
Sweden	2.8	1.8	2.2	2.9	3.3	3.9	3.9	2.7	5.1	4.4

**Figure 1.**
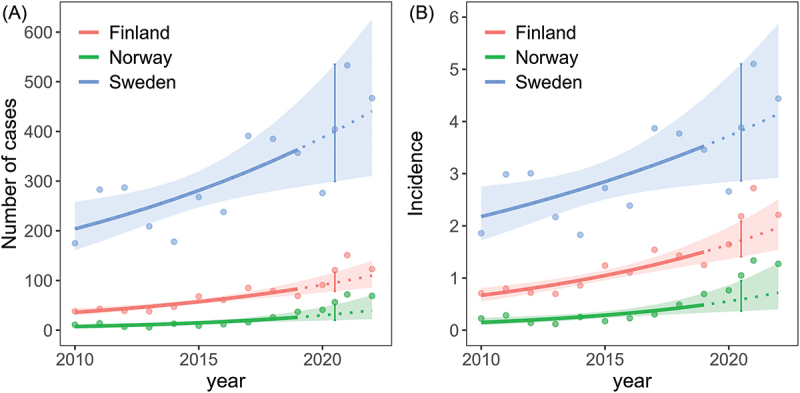
TBE (A) Annual reported cases and (B) Annual incidence per 100 000 inhabitants estimated as a function of year (linear at log-scale) for each country separately. The lines show mean estimates together with the 95% confidence envelops. Predictions for 2020–2022 (dashed lines) are based on the model fitted to the period 2010–2019. For each country, the average level of the pandemic years (2020–2021) are highlighted by error bars representing the estimate with 95% confidence intervals. Points show the raw data of reported cases per year (A) and annual incidence per 100 000 inhabitants (B).

For all the three countries there has also been an increase in reported cases over the whole study period (Supplementary Table S1). The predicted number of cases (and similarly, the incidence) in 2020–2022, based on a model fitted to the annual reported cases in 2010–2019, are shown in Figure 1. For the pandemic years together (2020/2021), the average annual counts were estimated to be 1.3 times higher for Finland (95% c.i.: 0.98, 1.6, *p* = 0.08), 1.7 times higher for Norway (95% c.i.: 0.94, 3.1, *p* = 0.08) and no difference for Sweden (95% c.i.: 0.63, 1.6, *p* = 0.97), compared to the expected sum of cases from the model accounting for an increasing trend over years (Figure 1, Supplementary table S2 and S3).

The predicted number of cases in 2020 was 91 (95% c.i.: 75.9, 109.6) and in 2021 100 (95% c.i.: 80.9, 123.8) compared to the reported 91 and 151 cases in Finland in 2020 and 2021, respectively. For Norway, the predicted number of cases in 2020 was 30 (95% c.i.: 19.4, 46.4) and in 2021 35 (95% c.i.: 20.8, 57.2) compared to the reported 41 and 72 number of cases in 2020 and 2021. For Sweden, the predicted number of annual cases in 2020 was 388 (95% c.i.: 296, 508) and in 2021 413 (95% c.i.: 303, 564) compared to the reported 276 (2020) and 533 (2021) annual cases. When considering the increasing trend of annual reported cases, the number of cases reported in 2020 were not higher than expected from the model, and for Sweden, the annual number tended to be 0.7 times lower than expected (95% c.i.; 0.5, 1.1, *p* = 0.13). For 2021, the annual sum of cases was estimated to be 1.5 times higher for Finland (95% c.i.: 1.2, 1.9, *p* = 0.001), 1.9 times higher than expected for Norway (95% c.i.: 1.2, 2.9, *p* = 0.003) and a tendence of 1.3 times higher for Sweden (95% c.i.: 0.8, 2.0, *p* = 0.27) compared to the expected annual sum of cases from the model accounting for an increasing trend over years. The same results were found when modelling the annual incidences (only minor changes in a few of the estimates).

### TBE seasonality

**Figure 2.**
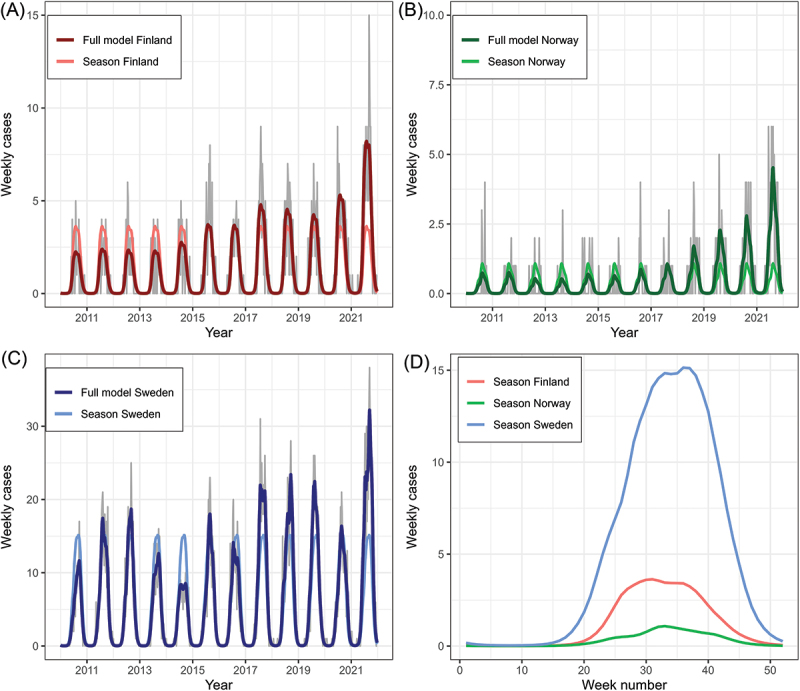
Weekly number of TBE cases fitted to a seasonal model for A) Finland, B) Norway and C) Sweden. The full model includes estimated values from both the seasonal part (common across years) and the long-term yearly trend of the model. Grey lines show the raw number of weekly cases. D) the seasonal part of the model extracted from each country.

When comparing weekly number of reported cases between Finland, Norway and Sweden, a distinct and similar seasonal trend and variation was found within and between the countries. The main seasonal trend across the study period (2010–2021), was similar between the three countries (Figure 2). The peak week of the seasonal trend fitted from the model was 31, 33 and 36, for Finland, Norway and Sweden, respectively. For Sweden, there was also a first (lower) peak in week number 33. For each of the three countries, the seasonal trend was higher or equal to the median for the week numbers 21–46.

The annual maximum number of reported cases per week (Supplementary Table S4) was higher for the pandemic years in total compared to the pre-pandemic years (Supplementary Table S5 and S6). For Finland, maximum number of cases per week ranged between 4 and 9 (mean = 6) for the period 2010–2019 and was significantly higher for the COVID-19 years (*p* = 0.004) with 9 cases in 2020 and 15 cases in 2021. For Norway, maximum number of cases per week ranged between 2 and 5 for the period 2010–2019 (mean = 3), compared to a peak of 4 cases in 2020 and 6 cases in 2021. The peak number in 2021 was significantly higher compared to peak numbers in 2010–2019 (*p* = 0.04). For Sweden, maximum number of cases per week ranged between 10 and 31 for the period 2010–2019 (mean = 22), compared to a peak number of 21 cases in 2020 and 38 cases in 2021. Compared to 2010–2019, the peak number of cases per week was significantly higher in 2021 (*p* = 0.04), but not for 2020 (*p* = 0.74). Although there was variation among years (Supplementary Figure S1), no strong trend in changes or differences in seasonality between the pre-pandemic years and the COVID-19 years were detected by descriptive summary statistics (Supplementary tables S4 and S5). The range of week numbers corresponding to the peak of weekly cases was 28–38 for Finland, 22–41 for Norway and 27–39 for Sweden. No differences were found in timing of the week with peak cases between 2010 and 2019 and the pandemic years.

The onset of TBE season in Norway in 2021 tended to start earlier than for the 2010–2019 period (*p* = 0.08). For the two other countries, we found no difference between the onset of TBE season during 2010–2019 and the years 2020–2021. The average week of onset during the years 2010–2019 was week 23, 30 and 18 for Finland, Norway and Sweden, whilst during the pandemic years the average onset was week 22, 22 and 19, respectively.

## Discussion

Human behaviour has shaped and spread infectious diseases for a millennia [72] and similarly COVID-19 mitigation measures has affected the incidence of many infectious diseases globally. This anthropause made it possible to quantify changes in human activities (mobility) on wildlife behaviour [73] and exemplified how human behavioural responses can constrain animal movement. In areas with lockdowns animals travelled longer distances compared to locations with less stringent measures [73]. Since wildlife serves as important reservoirs of zoonotic diseases, including TBE, the behavioural responses seen by these terrestrial mammals might also indirectly have impacted disease incidence.

The TBE incidence during the COVID-19 pandemic was estimated to be 1.3 times higher for Finland, 1.7 times higher for Norway but no difference for Sweden, when considering the already existing increasing trend of annual cases seen from 2010 to 2019. The COVID-19 pandemic profoundly impacted the outdoor recreation both in the Nordic countries and other parts of the world [74–77] and influenced the incidence of infectious diseases [54,60,78,79]. The timing of the initial shift in outdoor activities coincided with enforcement of stay-at-home orders, suggesting that changes in tick exposure were driven by COVID-19 behavioural changes. The TBE incidence is a function of risk (infected questing ticks), rate of human exposure, vaccination rate and the efficiency of the surveillance system. So human exposure (outdoor activity) and tick activity can be seen as prerequisites or drivers [13,34]. The TBEV lifecycle itself offers plenty of hypotheses and unanswered questions where presence/fluctuations of hosts and tick population dynamics seem to play an unclear role. The epidemiology/ecology behind TBE incidence is largely unknown but have been linked to interconnecting events; where wildlife, climate and environmental change together with socioeconomic factors are important [19,22,31,80].

Other factors that might also have impacted the long-term trend and annual variation in incidence counts in the longer run include vaccination uptake in the endemic areas, higher temperatures leading to prolonged tick activity (increased exposure time) and wider geographical distribution of *Ixodes*-ticks that has been observed in Fennoscandia [81–83], environmental factors, climate change and host animal population density/dynamics [20,84,85]. Additionally, over the past decades the diagnostics of TBE have improved and the general knowledge about tick-borne diseases has increased leading to increased recognition and reporting.

Why Southern Sweden has larger endemic areas and higher incidence compared to the more restricted focal-endemic areas in the southern-coastal areas of Finland and Norway is puzzling. However, the high-risk area in Sweden is also characterized by many lakes and being around big population centers near Stockholm which might possible be fueling the incidence. The population size in Sweden is around twice the neighboring countries Finland and Norway. As the COVID-19 pandemic restrictions were placed on international travel and visits to public venues, such as museums, training facilities, libraries; people in Finland, Norway and Sweden were all encouraged to spend more time outdoors. As an outcome, the citizens spent more time outdoors in general, and in their summerhouses/winter cottages especially [86]. In addition, as international travel decreased massively, there was an increase in national travel in all countries. In Norway, residents reported a record high amount of hiking during the pandemic [87]. A study from Oslo based on mobile tracking data showed that outdoor recreational activity increased by 291% during stay-at-home orders relative to a three-year average [88] and a recent published preprint found a nationwide increase in the use of green spaces in Norway during the COVID-19 pandemic [89]. Both pedestrians (walking, running, hiking) and cyclists appeared to have increased their activity. In Finland, a study about urban green infrastructure use showed that residents were more likely to visit urban green infrastructure (UGI) closer to their home during the pandemic compared with before the pandemic [90]. Another study from Finland using questionnaires showed that nearly half of the respondents increased their outdoor recreation and the majority of outdoor recreation sites were visited more or as often as before the pandemic [91]. The spatial analysis revealed that the most often visited recreation sites were near forests, in semi-natural areas and housing areas as well as relatively close to the respondent’s residence. More than half said they spent more time outdoors than they used to [91]. In Sweden, three studies showed increased participation in outdoor recreation. In a national survey, 50% of respondents stated increased frequency and 45% stated increased duration of outdoor time [92]. A regional study showed that more people (from 74% to 85%) were active outdoors at least once per week, often many times per week when comparing the time before the pandemic to the situation during the pandemic [92]. A local study produced similar trends and the analyses showed a significant difference between the amount of visits to nature before and during the pandemic [92]. All three studies showed changes in lifestyle and new outdoor routines and an additional study from the Stockholm area showed the same [92,93]. A global analysis [94] found that parks in most countries received more visitors; but with a huge variability. Green space use in Denmark increased up to 350 % whereas park visits in Sweden showed no change [94]. A cross-sectional study of UK adults [95] showed that 63% of the respondents decreased their time spent in green spaces following movement restrictions. Data from UK Google provide evidence that outdoor recreation patterns were significantly affected by the stay-at-home orders rules [75] and that greenspace use could in fact be seen to vary directly (and significantly from the baseline) accordingly to the strictness of stay-at-home orders policy and when restrictions were lifted. They found a negative correlation between strictness of restrictions and greenspace use.

Notably, there was no significant increase in TBE incidence during the pandemic years in Sweden, which relied more on voluntary action, urging personal responsibility instead of implementing restrictions, especially during the first year of the pandemic. During the second year of the pandemic in Sweden, the restrictions were more strictly enforced [96]. The question is if this also affected the degree or pattern of outdoor recreation during 2020 to a lesser extent than in the neighbouring countries. A global analysis showed that park visits in Sweden did not significantly change whilst for Denmark, park visits increased continuously during the pandemic (and increased by up to 350%) [94]. It is interesting that we found a tendence for decreased incidence in Sweden for 2020 (0.7 times lower than expected (95%c ci: 0.5,1.1)) whilst for 2021, the incidence was higher, but not significantly different from the expected incidence from the model accounting for an increasing trend over years. A limitation of the mobility studies is that they might possibly not reflect changes in the specific areas with TBE disease registrations, which are known to have a strong focal distribution [97,98].

The pandemic has underlined the importance of outdoor space. Before the pandemic just 15% of the work forcein the EU had ever teleworked [65]. According to a post-COVID-19 global survey by Global Workplace Analytics, 94% of workers say they want to work from home at least occasionally in the future. Hybrid models of remote work persist in the wake of the pandemic. The virus has broken through cultural and technological barriers in terms of where work takes place. A McKinsey survey of office space managers conducted in 9 countries found that after the pandemic, they expected a 36% increase in worktime outside their offices, affecting main offices and satellite locations [66]. The results of this study indicate that the activities with the biggest appeal, and most likely to be long-lasting, are teleworking, spending more time outdoors and increasing travelling domestically. Several papers have pointed out that the recreational behavioral changes seen would also persist after the pandemic [68,99,100] and have identified an ‘outdoorification’ process that indicate an increase in participation and diversification of outdoor activity that will continue after the pandemic. A review paper [101] concluded that a high visitation rate in natural environments will remain and is likely to sum up to 30–40% more use than before the pandemic [101,102]. Google mobility data from Finland, Norway and Sweden also show a relatively sustained high rate of outdoor visitations after the pandemic (See Supplementary Figure S2).

The TBE cases show a strong and concordant seasonality. When comparing weekly number of cases between Finland, Norway and Sweden, a distinct and similar seasonal trend and variation was found between and within the countries (Figure 2, Supplementary Figure S1). The peak number of cases per week was significantly higher in 2021 (compared to 2010–2019) for all the three countries even though no differences were found in timing of peak cases between 2010 and 2019 and the pandemic years. The onset of the TBE season in Norway started earlier in 2021 compared to the 2010–2019 period. For the two other countries, we found no difference in the onset of TBE season during 2010–2019 and the pandemic years. A recent study was the first in Europe to detect changes in the seasonality of Lyme borreliosis [70]. This highlights the potential for climate and environmental change to shape the seasonal dynamics of vector-borne disease systems. In Germany, they found evidence of a shift of the TBE season by approximately 12 days over a 18-year period [61]. Little is known about the changing seasonality of human infections with TBEV in the wake of the ongoing climate and environmental change.

## Conclusion

We found a higher increase than expected in TBE cases during the pandemic years in Finland and Norway. However, in Sweden, the increase in cases was as predicted. In addition, we saw an increasing trend of TBE in all three Nordic countries from 2010 to 2021.

The complexity, severity and consequences of the COVID-19 restrictions in terms of societal well-being and overall disease burden have not yet been fully understood and documented. The link between outdoor recreation and wellbeing is well-established and particularly during the pandemic this became evident. There is a need of increased awareness that public health interventions that are designed to mitigate against specific pandemic diseases need to be balanced with possible increased risk for other diseases and overall disease burden.

We recommend developing effective communication material to be distributed through carefully chosen channels to raise awareness about tick-borne diseases in situations when people’s movement is restricted, and outdoor activity is encouraged. Proportioned risk perception and knowledge about protective measures is needed to prevent tick-borne diseases.

## Supplementary Material

Supplemental Material

## Data Availability

Available on the public health institutes webpages
