# Supplementary material for: Outdoor recreation, tick borne encephalitis incidence and seasonality in Finland, Norway and Sweden during the COVID-19 pandemic (2020/2021)
Source: Infect Ecol Epidemiol. 2023 Nov 18;13(1):2281055. doi: 10.1080/20008686.2023.2281055 (PMC10769561; doi:10.1080/20008686.2023.2281055)

Supplemental material

**Outdoor recreation, tick borne encephalitis incidence and seasonality in Finland, Norway and Sweden during the COVID-19 pandemic (2020/2021)**

Solveig Jore, Hildegunn Viljugrein, Marika Hjertqvist, Timothée Dub and Henna Mäkelä

Supplementary Table S1. Negative binomial regression of annual number of cases for the pre-pandemic period (2019-2019) as a function of year. The exponential of the year coefficient show the estimated mean rate of increase in (a) annual reported cases or (b) annual incidence. b) For modelling annual incidence as function of year, log-population size is included as an offset

|  | Intercept^1^ | SE | year | year_SE | exp(year) | p-value | ΔAIC^2^ |
| --- | --- | --- | --- | --- | --- | --- | --- |
| 1. *Annual number of cases* | | | | | | | |
| Finland | 4.049 | 0.047 | 0.093 | 0.017 | 1.10 | <0.001 | -12.8 |
| Norway | 2.696 | 0.112 | 0.141 | 0.040 | 1.15 | <0.001 | -6.4 |
| Sweden | 5.639 | 0.065 | 0.064 | 0.022 | 1.07 | 0.004 | -4.2 |
| 1. *Annual incidences* | | | | | | | |
| Finland | -11.467 | 0.047 | 0.090 | 0.017 | 1.09 | <0.001 | -12.3 |
| Norway | -12.757 | 0.113 | 0.130 | 0.040 | 1.14 | 0.001 | -5.4 |
| Sweden | -10.467 | 0.065 | 0.054 | 0.022 | 1.06 | 0.016 | -2.7 |

^1^The intercept refers to year=2015.

^2^ΔAIC shows the improvement in AIC by including year in the model (compared to a model with intercept only).

Supplementary Table S2. Reported number of (a) TBE cases or (b) incidence (per 100 000) compared to predicted number of cases or incidence for post-pandemic (2022) and pandemic years (2020-2021) from the regression model of the pre-pandemic period (see Supplementary Table S2). The comparison of annual cases (incidence) against predicted numbers is also shown in Figure 1 of this paper.

| Country | Year^1^ | incidence | Predicted | Pred_low | Pred_high |
| --- | --- | --- | --- | --- | --- |
| 1. *Annual number of cases* | | | | | |
| Finland | 2020 | 91 | 91.211 | 75.897 | 109.614 |
| Finland | 2021 | 151 | 100.082 | 80.914 | 123.791 |
| Finland | 2020.5 | 121 | 95.544 | 78.377 | 116.47 |
| Finland | 2022 | 123 | 109.816 | 86.18 | 139.935 |
| Norway | 2020 | 41 | 29.977 | 19.388 | 46.35 |
| Norway | 2021 | 72 | 34.516 | 20.837 | 57.173 |
| Norway | 2020.5 | 56.5 | 32.166 | 20.106 | 51.46 |
| Norway | 2022 | 69 | 39.741 | 22.344 | 70.685 |
| Sweden | 2020 | 276 | 387.65 | 295.783 | 508.051 |
| Sweden | 2021 | 533 | 413.381 | 303.195 | 563.611 |
| Sweden | 2020.5 | 404.5 | 400.309 | 299.513 | 535.026 |
| Sweden | 2022 | 467 | 440.82 | 310.469 | 625.9 |
| 1. *Annual incidences* | | | | | |
| Finland | 2020 | 1.644 | 1.641 | 1.367 | 1.971 |
| Finland | 2021 | 2.722 | 1.796 | 1.453 | 2.22 |
| Finland | 2020.5 | 2.184 | 1.717 | 1.409 | 2.091 |
| Finland | 2022 | 2.211 | 1.964 | 1.543 | 2.501 |
| Norway | 2020 | 0.764 | 0.553 | 0.356 | 0.86 |
| Norway | 2021 | 1.335 | 0.63 | 0.378 | 1.051 |
| Norway | 2020.5 | 1.05 | 0.591 | 0.367 | 0.95 |
| Norway | 2022 | 1.272 | 0.718 | 0.401 | 1.286 |
| Sweden | 2020 | 2.659 | 3.722 | 2.843 | 4.871 |
| Sweden | 2021 | 5.104 | 3.927 | 2.884 | 5.346 |
| Sweden | 2020.5 | 3.885 | 3.823 | 2.864 | 5.102 |
| Sweden | 2022 | 4.439 | 4.143 | 2.923 | 5.872 |

^1^Year 2020.5 represents the average level of the pandemic years (2020-2021)

Supplementary Table S3. Negative binomial regression of annual (a) number of cases and (b) incidence for the pre-pandemic period (2019-2019 and covid-19 average) as a log-linear function of year and a categorical variable (2020/2021) identifying the pandemic-years. The exponential of the pandemic-year estimate show the estimated mean rate of increase in (a) annual reported cases or (b) annual incidence relative to the increase expected from the log-linear trend over years.

|  | Intercept^1^ | SE | year | year_SE | 2020/2021 | 2020/2021_SE | exp(2020/2021) | p-value |
| --- | --- | --- | --- | --- | --- | --- | --- | --- |
| 1. *Annual number of cases* | | | | | | | | |
| Finland | 4.049 | 0.044 | 0.092 | 0.015 | 0.238 | 0.134 | 1.3 | 0.08 |
| Norway | 2.695 | 0.100 | 0.146 | 0.036 | 0.529 | 0.302 | 1.7 | 0.08 |
| Sweden | 5.639 | 0.062 | 0.064 | 0.021 | 0.009 | 0.237 | 1.0 | 0.97 |
| 1. *Annual incidences^2^* | | | | | | | | |
| Finland | -11.47 | 0.043 | 0.090 | 0.015 | 0.243 | 0.133 | 1.3 | 0.07 |
| Norway | -12.76 | 0.102 | 0.135 | 0.036 | 0.543 | 0.309 | 1.7 | 0.08 |
| Sweden | -10.47 | 0.062 | 0.054 | 0.021 | 0.015 | 0.235 | 1.0 | 0.95 |

^1^The intercept refers to year=2015.

^2^For modelling (b) annual incidence as function of year, log-population size is included as an offset

**Seasonal TBE data: summary of weekly reported TBE cases**

Supplementary Table S4. Weekly reported TBE cases summarized for the pre-pandemic and pandemic years by a) annual onset of the TBE season defined by the first week for which there was at least 2 reported cases for current and previous week in total, b) annual peak number of cases and c) annual week number corresponding to the first week with peak number of cases.

|  | Pre-pandemic years 2010-2019 | | | | |  |  | COVID-19 years | |  |
| --- | --- | --- | --- | --- | --- | --- | --- | --- | --- | --- |
| Country | Mean | Min | 25 % | 50 % | 75 % | Max |  | Mean | 2020 | 2021 |
| *a) Onset of the TBE season* | | | | | | | | | | |
| Finland | 23 | 20 | 22 | 23 | 24 | 26 |  | 22 | 22 | 22 |
| Norway | 30 | 22 | 24.8 | 31 | 32.8 | 41 |  | 21.5 | 25 | 18 |
| Sweden | 18.4 | 14 | 18 | 18 | 18.8 | 23 |  | 19 | 20 | 18 |
| *b) The peak number of weekly reported cases* | | | | | | | | | | |
| Finland | 6 | 4 | 5 | 5.5 | 7 | 9 |  | 12 | 9 | 15 |
| Norway | 3.1 | 2 | 2 | 3 | 4 | 5 |  | 5 | 4 | 6 |
| Sweden | 21.7 | 10 | 17.8 | 22 | 25.8 | 31 |  | 29.5 | 21 | 38 |
| *a) Week number corresponding to the first week with peak number of cases* | | | | | | | | | | |
| Finland | 31.2 | 28 | 30 | 31 | 31.8 | 35 |  | 31 | 26 | 36 |
| Norway | 33.8 | 22 | 32 | 34 | 37.5 | 41 |  | 27.5 | 31 | 24 |
| Sweden | 33.4 | 27 | 30.3 | 33.5 | 37.5 | 38 |  | 35.5 | 34 | 37 |

Supplementary table S5. Results from regression models (mean estimates, standard errors and p-values) testing differences between pre-pandemic years (2010-2019) and COVID-19 years in a) onset of the TBE season, b) the peak number of weekly reported cases and c) week number corresponding to the peak. COVID-19 years were included as a covariate (2020/2021).

| Country | Intercept | Intercept_SE | 2020/2021 | 2020/2021_SE | p-value |
| --- | --- | --- | --- | --- | --- |
| *a) Onset of the TBE season* | | |  |  |  |
| Finland | 3.135 | 0.025 | -0.044 | 0.061 | 0.486 |
| Norway | 3.401 | 0.062 | -0.333 | 0.174 | 0.085 |
| Sweden | 2.912 | 0.039 | 0.032 | 0.095 | 0.743 |
| *b) The peak number of weekly reported cases* | | | |  |  |
| Finland | 1.792 | 0.099 | 0.693 | 0.184 | 0.004 |
| Norway | 1.131 | 0.112 | 0.478 | 0.228 | 0.062 |
| Sweden | 3.077 | 0.099 | 0.307 | 0.213 | 0.181 |
| *a) Week number correpsonding to the first week with peak number of cases* | | | | | |
| Finland | 3.44 | 0.032 | -0.006 | 0.078 | 0.936 |
| Norway | 3.52 | 0.049 | -0.206 | 0.132 | 0.149 |
| Sweden | 3.51 | 0.037 | 0.061 | 0.089 | 0.511 |

Supplementary table S6. Results from regression models testing differences between pre-pandemic years (2010-2019) and COVID-19 years in a) onset of the TBE season, b) the peak number of weekly reported cases and c) week number corresponding to the peak. COVID-19 years were included as covariates for separate years (2020 and 2021).

| Country | y2020 | y2021_SE | p-value | y2021 | y2021_SE | p-value |
| --- | --- | --- | --- | --- | --- | --- |
| *a) Onset of the TBE season* | | |  |  |  |  |
| Finland | -0.044 | 0.088 | 0.625 | -0.044 | 0.088 | 0.625 |
| Norway | -0.182 | 0.222 | 0.433 | -0.511 | 0.259 | 0.080 |
| Sweden | 0.083 | 0.130 | 0.537 | -0.022 | 0.136 | 0.875 |
| *b) The peak number of weekly reported cases* | | | |  |  |  |
| Finland | 0.405 | 0.248 | 0.137 | 0.916 | 0.2 | 0.001 |
| Norway | 0.255 | 0.332 | 0.462 | 0.66 | 0.279 | 0.042 |
| Sweden | -0.033 | 0.307 | 0.917 | 0.56 | 0.236 | 0.042 |
| *a) Week number correpsonding to the first week with peak number of cases* | | | | | |  |
| Finland | -0.182 | 0.084 | 0.058 | 0.143 | 0.072 | 0.080 |
| Norway | -0.086 | 0.169 | 0.622 | -0.342 | 0.191 | 0.106 |
| Sweden | 0.018 | 0.128 | 0.893 | 0.102 | 0.123 | 0.428 |

**Supplementary Figure S1**

Supplementary Figure S1. Seasonal pattern of TBE weekly cases for the three countries shown as times series of raw case numbers for the 12 years 2010-2021 (A-C).


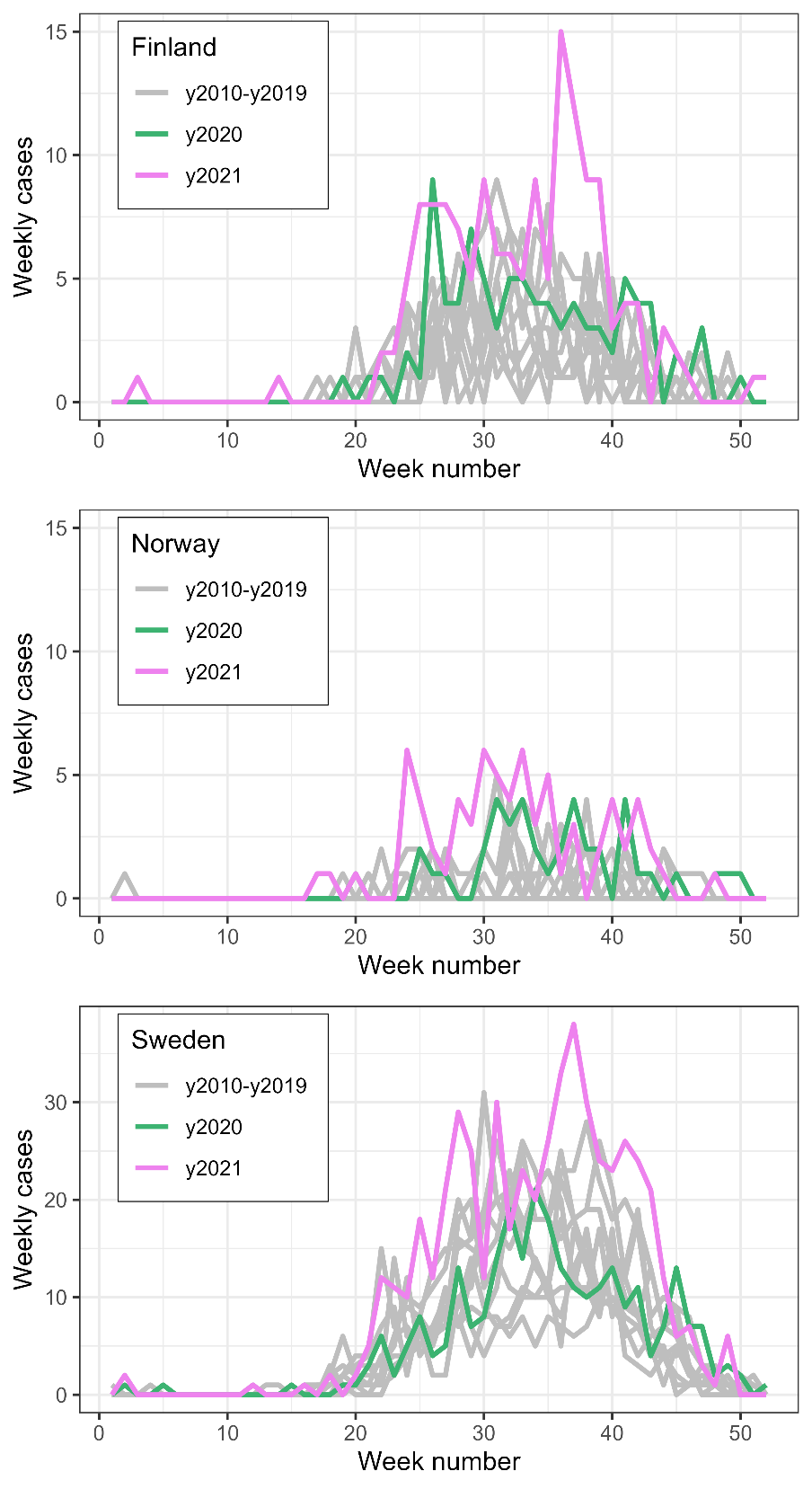


**Supplementary Figure S2**

[Google Mobility Trends: How has the pandemic changed the movement of people around the world? - Our World in Data](https://ourworldindata.org/covid-mobility-trends)


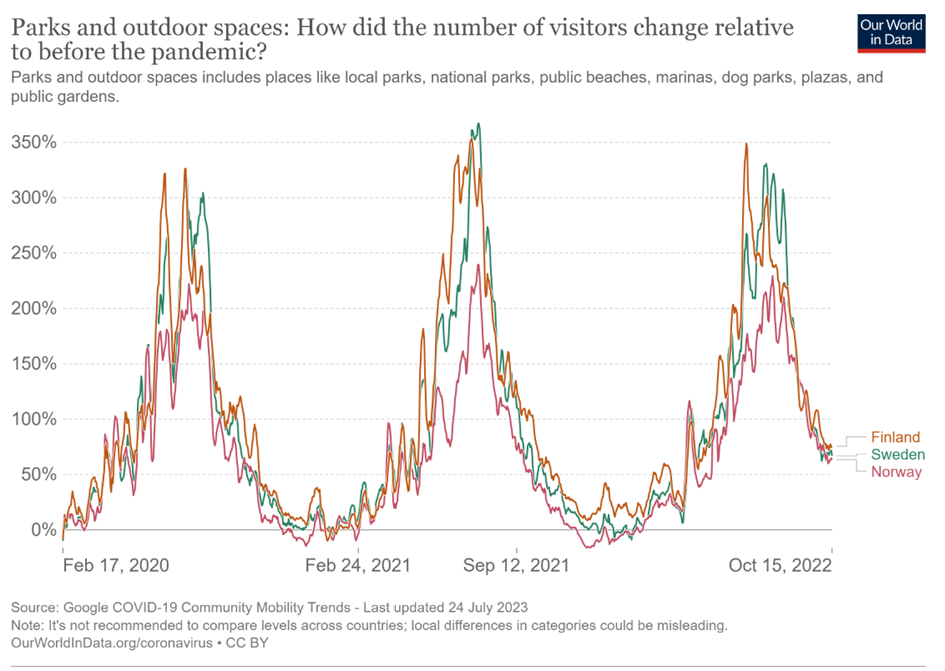


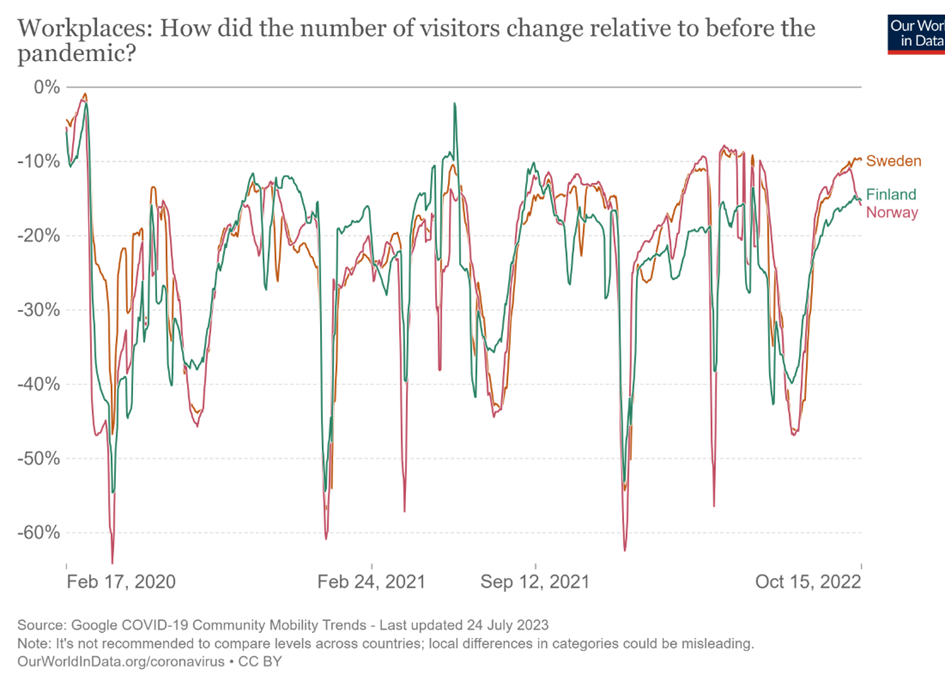

Supplement: Supplemental Material [file ZIEE_A_2281055_SM4145.docx]
